# Integrating DNA-Based Memory in Water-Resistant Electrospun Polymer Fibers for Nondestructive Data Retrieval

**DOI:** 10.1021/acsami.5c06554

**Published:** 2025-07-30

**Authors:** Cecilia Wetzl, Diana Soukarie, Jokin Yeregui Elosua, Ibon Santiago

**Affiliations:** † 138823 CIC nanoGUNE BRTA, 20018 Donostia-San Sebastián, Spain; ‡ University of the Basque Country, UPV/EHU, 20018 Donostia-San Sebastián, Spain

**Keywords:** DNA data storage, water-resistant, electrospinning, fibers, DNA preservation

## Abstract

DNA is a digital memory storage medium with advantageous properties, including longevity and high information density. Embedding information-bearing oligonucleotides into materials for long-term storage has gained traction by leveraging modern coding, DNA synthesis, and sequencing technologies. Here, we present a versatile way to store digital information in synthetic DNA embedded in polymer fibers. These composite fibers are made of hydrophilic (poly­(vinyl alcohol) and poly­(ethylene oxide)) and hydrophobic (polycaprolactone and cellulose acetate) polymers synthesized by solution electrospinning and followed by cross-linking to enhance water resistance. We demonstrate the on-demand retrieval from all fiber compositions of short and long messages encoded in a single oligonucleotide and a pool of oligonucleotides, respectively. DNA/cellulose acetate fiber composites are true nondestructive readout memory: repeated access to messages stored in fibers is afforded without damaging the integrity of fibers or DNA. We envisage that our simple and robust manufacturing approach will contribute to the development of scalable and accessible DNA data storage solutions.

## Graphical abstract

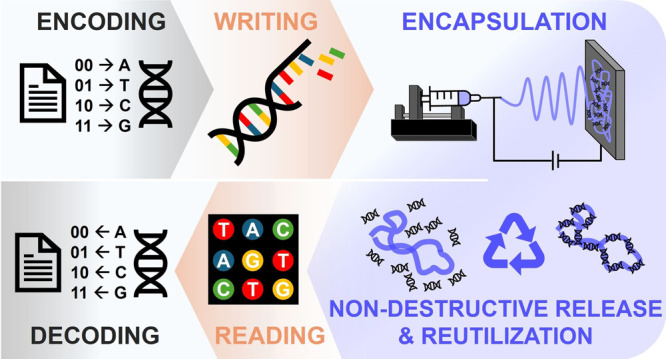

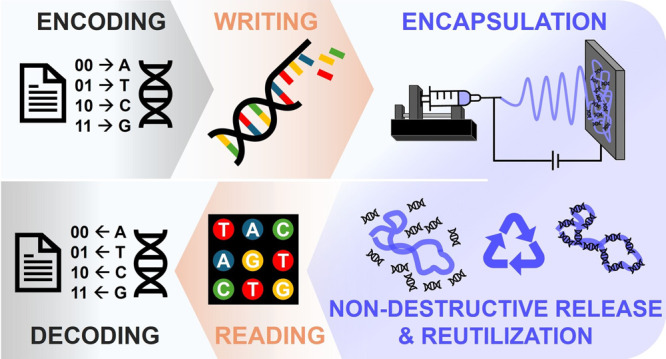

## Introduction

The exponential growth in data generation has become a critical challenge for memory storage systems. By the end of 2025, the volume of data is expected to reach 175 Zettabytes worldwide,^1^ mainly due to the spread of the Internet of Things (IoT) devices and the construction of big data sets for cloud storage and artificial intelligence applications.^2–4^ Over the last 15 years, digital data storage has expanded beyond conventional physical media like magnetic hard disk drives (HDDs), optical discs, and solid-state flash memory. Specifically, research in memory storage focuses on optimizing features such as information density, access rates, ultralong archival times, and energy consumption, which are shared metrics in state-of-the-art storage approaches.^5,6^ The employment of innovative storage media such as phase-change materials,^7^ liquid memory,^8^ nanostructured glass,^9^ and 2D materials^10^ exhibited promising performances for the development of next-generation storage systems. In this race, nature provides an exceptional medium for digital data storage: DNA. DNA offers superior advantages such as high information density (up to 250 petabytes per gram)^11^ and centuries-long, energy-efficient data retention.^12,13^ Thanks to advances in data-to-DNA encoding schemes,^11,14,15^ together with the development of next-generation synthesis^16^ and sequencing^17,18^ methods, DNA-based solutions have proven to be a reliable option for the future of massive digital data storage. In this context, DNA dry preservation systems have emerged as a viable method, as they do not require refrigeration, thereby enhancing cost-effectiveness and reducing environmental impact. To ensure the stability of dry DNA for long-term archival storage, embedding it in a robust matrix is essential.^13^

Encapsulation in silica beads was one of the first methods proposed to preserve DNA in the dry state, because it uses natural fossilization-like processes to shield DNA from deterioration and environmental harm.^19–21^ While this method showed outstanding stability, it requires harsh chemical treatments (e.g., buffered hydrofluoric acid) to access the DNA, making it unsuitable for large-scale applications and nondestructive readout. Recently, the use of polymeric matrices has emerged as a valuable alternative for embedding DNA to enhance preservation. Specifically, both synthetic^22–24^ and naturally derived polymers—such as silk fibroin^25^—have been employed for the mid- and long-term storage of both naked and protected DNA (i.e., secured within vesicles or beads), enabling fast access to the information (either genetic or digital) using milder retrieval conditions. In this fashion, we previously demonstrated the effective encoding and retrieval of a short message into naked DNA embedded in polymeric nanofibers produced by electrospinning and melt-electrowriting methods.^26^ In our system, solution-electrospinning from water or acetone blends enabled the creation of homogeneous nanofibers by directly mixing the target DNA with the polymeric matrix solution. The use of nanofibers as an embedding matrix for DNA provides significant protection against environmental stresses^13^ while offering an effective DNA retrieval method through fiber dissolution within a few minutes. Herein, we developed an end-to-end workflow from data-to-DNA message encoding, preservation in polymeric fibers, and DNA-controlled release, followed by DNA-to-data decoding (Figure 1). We explored and built a library of electrospinnable polymers compatible with DNA encapsulation for mid/long-term storage, opening the door to further investigations into polymer-based DNA dry storage systems. To achieve high DNA protection against environmental factors (e.g., humidity and temperature), our investigation focused on polymeric fibers produced from either hydrophobic or hydrophilic polymers with increased water resistance achieved through cross-linking strategies. In addition to high humidity and temperatures, nucleases and the generation of reactive oxygen species (ROS) are important risk factors affecting DNA integrity. However, several studies demonstrated the functional integrity of plasmid DNA or siRNA embedded into electrospun fibers for genetic material delivery,^27^ reinforcing the hypothesis that fiber encapsulation can provide physical protection against these factors. With our newly designed experiments, we studied their resistance to high humidity and high temperatures thoroughly, as these environmental conditions can induce DNA damage. Water resistance is especially relevant for producing reusable matrices that can release incorporated DNA on demand while preventing the destruction of fiber meshes when retrieving the information and keeping the encoded data intact for future use.

**Figure 1:**
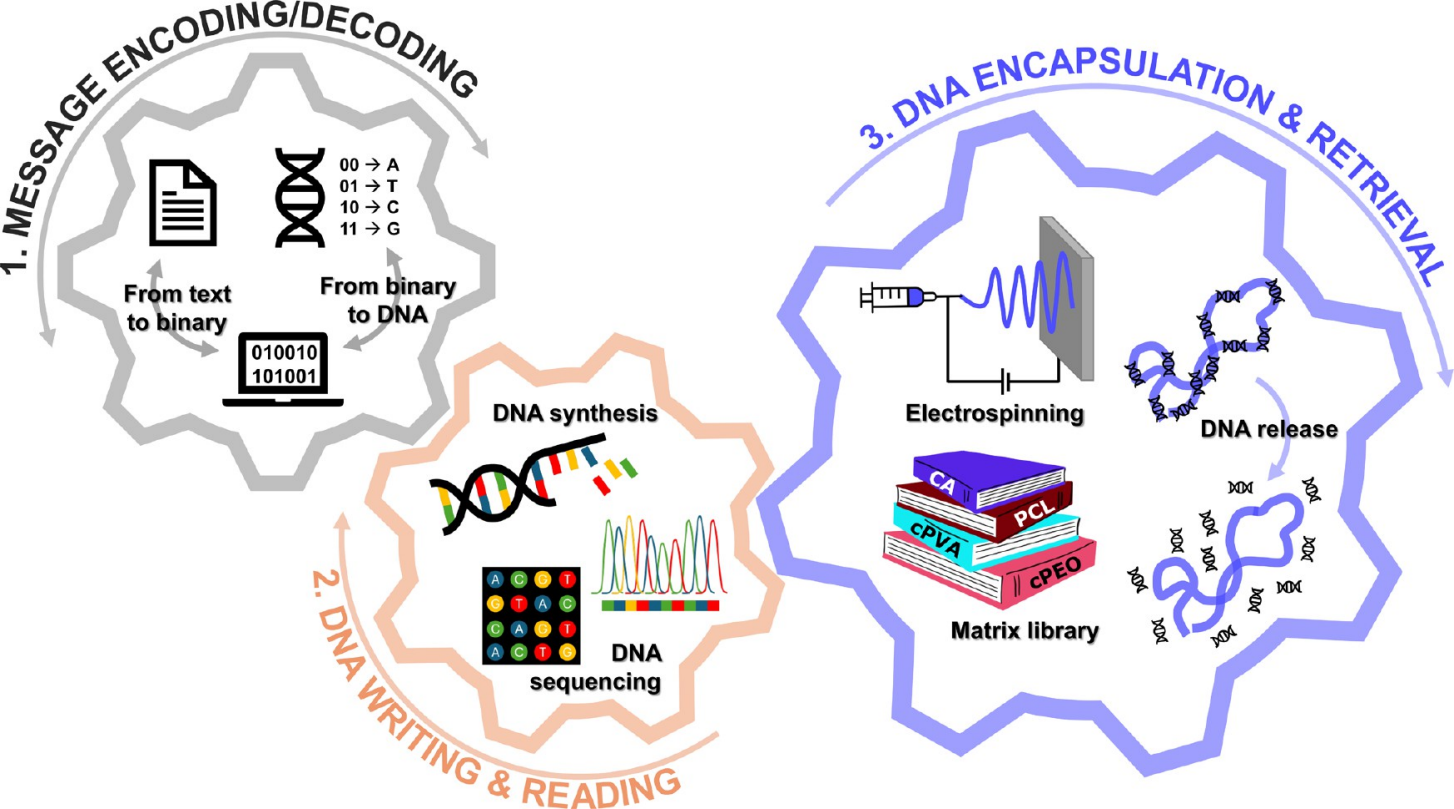
Schematic overview of the experimental workflow, starting with message encoding (1) and DNA synthesis (2), followed by matrix selection and fiber formation (3); DNA is subsequently retrieved through release from the fiber, sequencing (2) and decoding (1).

Our formulations displayed remarkable resistance to environmental factors, without compromising the facile retrieval of DNA, which is triggered on demand through solvent addition and heating. We demonstrated that storage within polymeric fibers preserves the DNA structure, which, upon release, is ready for PCR amplification and sequencing, allowing the decoding of complex messages encoded in a DNA oligonucleotide pool (DOP). Our method represents a promising approach for DNA digital data storage with potential applications in “DNA-of-things”, including its use in the textile industry.^20^ Incorporating metadata-embedding nanofibers into fabrics is of great interest for enabling complete traceability of textile-like products. These applications can help combat counterfeiting and address environmental and ethical concerns in the sector, while offering a record of a product lifecycle through a DNA-based digital product passport (DPP).^28^

## Results and Discussion

### DNA Encapsulation in Polymeric Fibers

Polymeric fibers containing DNA were produced by solution electrospinning following the workflow described in the Materials and Methods section. First, the DNA/polymer mixture was prepared in a DNA-compatible solvent (water or acetone) for four different formulations: (i) 25% w/v polycaprolactone (PCL), (ii) 10% w/v cellulose acetate (CA), (iii) 8% w/v poly­(vinyl alcohol) (PVA), and (iv) 8% w/v poly­(ethylene oxide) (PEO). Due to the premixing procedure, the encapsulation protocol is irreversible. Fiber meshes were produced using optimized parameters for each polymer solution (see Materials and Methods). While PCL and CA are *ready to use* after electrospinning, PVA and PEO were subjected to an additional cross-linking step to enhance their stability under ambient conditions. In fact, the complete and fast dissolution of hydrophilic fibers in high humidity makes them unsuitable for long-term data storage. The selected cross-linking method for PVA meshes was a glutaraldehyde (GA) vapor exposition for 48 h. This is a common method employed in reticulation of hydroxyl-bearing polymers due to their chemical structure, which can react with GA, bridging parallel polymer chains.^29,30^

For PEO meshes, another widely used method for cross-linking was used, namely, light-activated cross-linking using a reticulating agent and a photoinitiator as additives. Here, PEO was mixed with trimethylolpropane triacrylate (TMPTA) and benzophenone before the electrospinning step. Once the fiber mesh was formed, it was subjected to 254 nm UV irradiation for 4 h to trigger the radical reaction between the TMPTA chains, producing a high degree of reticulation.

The meshes were morphologically characterized by scanning electron microscopy (SEM) (Figure 2). The mean fiber diameter was calculated to be 207.3 ± 35.2 nm for cross-linked PVA (cPVA), 438.5 ± 62.3 nm for cross-linked PEO (cPEO), 502.3 ± 94.1 nm for PCL, and 448.2 ± 74.3 nm for CA. The SEM images showed good homogeneity for cPVA, cPEO, and CA, whereas PCL showed a partially heterogeneous fiber mesh composed of smaller fibers with a diameter around 450 nm and some bigger fibers of 650 nm (results summarized in Figure S1).

**Figure 2:**
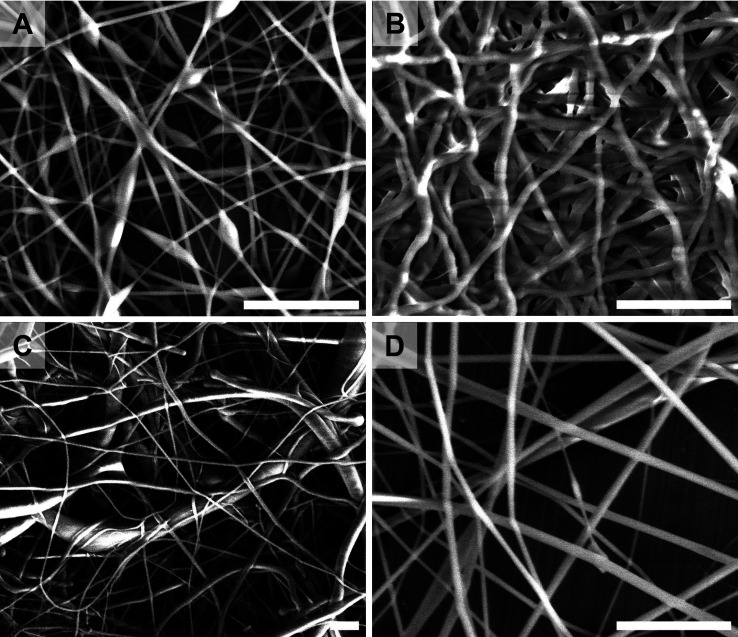
SEM characterization of polymeric fibers of different composition: (a) cPVA, (b) cPEO, (c) PCL, and (d) CA. All the polymeric fibers contain 0.0034% w/w of message-encoded DNA. The scale bar is 5 μm.

### Controlled DNA Release

Once the fiber mesh is prepared, the next steps in the workflow involve releasing the DNA in a controlled manner and accessing the stored information. As a proof of concept, we encapsulated a small text message (“Nanogune”) encoded in a 111-base long oligonucleotide, called *Mezu* hereafter, in the four polymeric formulations described above. In this case, we used a simple encoding scheme^26^ and the message was repeated twice to limit information loss during the decoding process. The DNA release was induced by soaking the polymeric mesh in water or acetone. The samples were subjected to PCR amplification, and the presence of *Mezu* was evaluated through gel electrophoresis (GE).

To confirm that cross-linking does not affect DNA integrity while still enhancing the shelf life of the sample, we compared the release of DNA from the PEO and PVA fiber meshes with their cross-linked counterparts. Additionally, for all the samples, PCR amplification was performed on a 5 μL sample collected immediately after water immersion of the fibers at rt and after their complete dissolution by heating at 80 °C. This experiment helped determine whether it is necessary to destroy the fiber structure through dissolution to release DNA, or if its passive diffusion in a solvent is sufficient to retrieve the message. As shown in Figure 3A,B for both PEO and PVA, a clear single band corresponding to the PCR amplified *Mezu* was present for all samples observed on agarose GE, except in the negative controls. This result confirmed that the cross-linking protocols employed are not affecting the recovery of DNA. Additionally, the DNA was released from samples solubilized at rt and at 80 °C, indicating that, through water immersion, DNA diffuses passively from the fibers without the need for complete denaturation of the fiber structure. It is worth noting that while PEO and PVA were partially solubilized at rt, cPVA and cPEO samples showed integrity of the mesh soaked in water prior to heating at 80 °C, reinforcing the observation that the fiber structure can be preserved while still allowing access to the DNA molecules.

**Figure 3:**
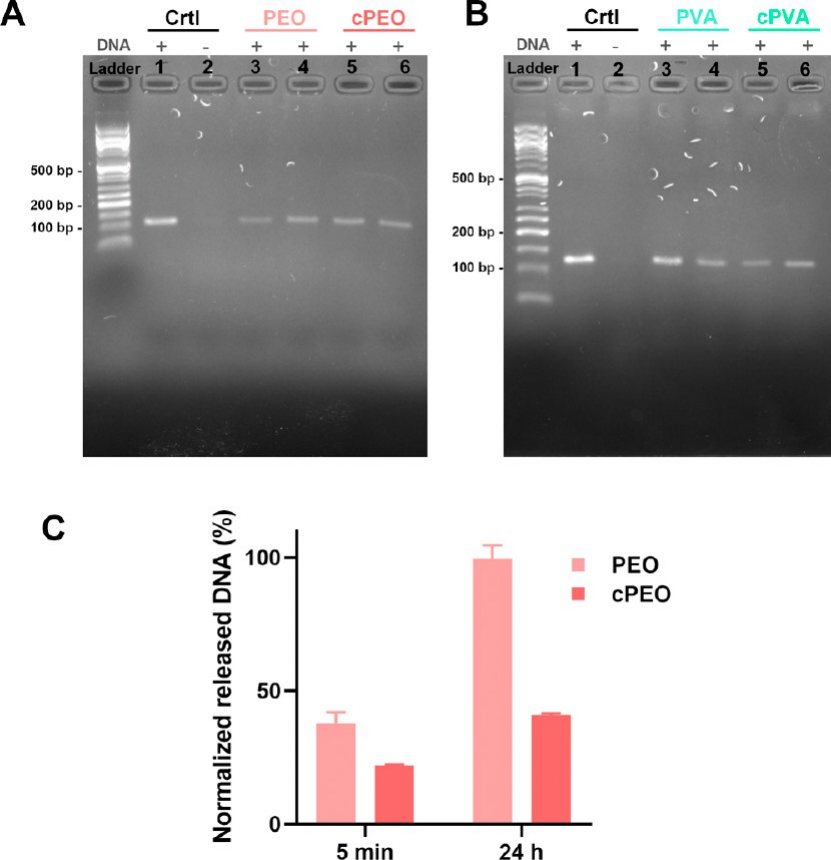
DNA released from hydrophilic fibers. (A,B) Agarose GE after PCR amplification of the original reference DNA sequence (lanes 1 and 2) and DNA sequence retrieved from PEO and cPEO (A) or PVA and cPVA (B) through solubilization at room temperature (lanes 3 and 5) or at 80 °C (lanes 4 and 6). DNA appears as a distinct band in the samples containing DNA (marked as + ), while no nonspecific amplification is detected when no reference DNA is present in the samples (−). (C) DNA release measured by UV–vis absorption at 260 nm after 5 min and 24 h of soaking in water for PEO and cPEO. The results are expressed as average ± standard deviation (*n* = 3). The absorbance values are normalized to the maximum amount of released DNA after complete dissolution.

To gather more information about DNA release, the DNA amount released from the polymeric fiber when soaked in water was directly measured through UV–vis spectroscopy based on the absorbance of the sample at 260 nm, typically used to quantify double and single-stranded DNA in solution. In this case, the absorbance of the solution was analyzed over time, comparing the quantity of DNA released after 5 min and 24 h in solution. The absorbance of each sample was normalized to the maximum absorbance obtained after the complete dissolution of the fibers, induced upon heating of the sample to 80 °C. Due to a partial overlap between the polymer and the DNA absorption spectra, quantification was not performed for PVA (Figure S2).

Comparing the cross-linked samples with their noncross-linked analogues, a decrease in the amount of released DNA after 5 min and 24 h confirmed the efficacy of the cross-linking for cPEO (Figure 3C). Indeed, 5 min after soaking the fibers in water, cPEO released 16% less DNA relative to PEO. After 24 h, a complete release was noticed for PEO, while cPEO showed a decreased release of 40.9%. This result confirmed that cross-linking increases the water resistance of the hydrophilic fibers by decreasing their solubility and slowing the DNA release over time.

As performed for hydrophilic polymers, we evaluated the DNA release from hydrophobic polymers, namely, PCL and CA. GE performed after PCR amplification of acetone-dissolved samples showed a clear band for both PCL and CA fibers with embedded *Mezu* DNA, which was absent in the negative control samples (Figure 4A). Amplified *Mezu* also appeared as a distinct band in the sample prepared by soaking the hydrophobic fibers in water (Figure S3). From the UV–vis absorption at 260 nm, it was observed that for both PCL and CA only a minimal amount of the total embedded DNA (12.2% and 20.8%, respectively) is released in water, even after 24 h of incubation (shown in Figure 4B). This confirms that the DNA can diffuse in solution even without disrupting the fiber structure, but its release is limited and most of the sample is still preserved in the fibers.

**Figure 4:**
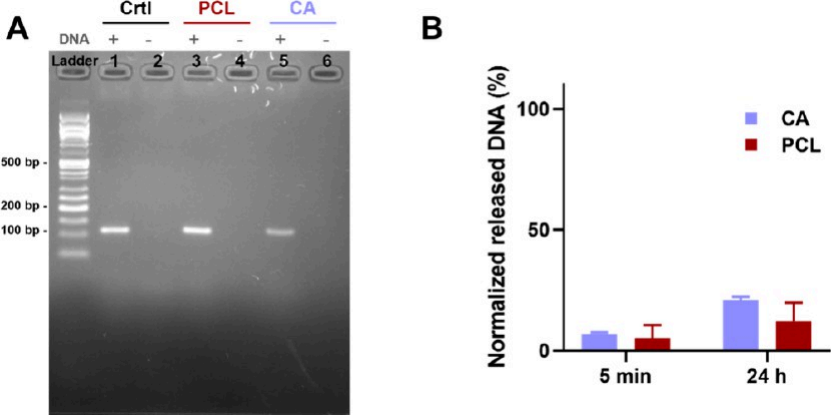
DNA release from hydrophobic fibers. (A) Agarose GE after PCR amplification of original reference DNA sequence (lanes 1 and 2) and DNA sequence retrieved from PCL (lanes 3 and 4) and CA (lanes 5 and 6) fibers through solubilization in acetone. DNA appears as a distinct band in the samples containing DNA (marked as + ), while no nonspecific amplification is detected when no reference DNA is present in the samples (−). (B) DNA release measured by UV–vis absorption at 260 nm after 5 min and 24 h of soaking in water. The results are expressed as average ± standard deviation (*n* = 3). The absorbance values are normalized to the maximum amount of released DNA after complete dissolution.

To further confirm that our storage methodology was effective and not damaging for the embedded message, the DNA samples extracted from the four different matrices were sequenced using the Sanger method. Sequencing results showed the message alignment and recovery in its second iteration for all of the samples (Figure S4).

### Fiber Stability and Accelerated Aging

The newly produced DNA-embedded fiber meshes were exposed to high humidity and high temperature to test their stability under extreme environmental conditions. High humidity exposure was performed using an environmental SEM (ESEM), equipped with a sample holder with temperature control, which allowed the relative humidity values to reach up to 100% by increasing the chamber pressure at a sample temperature of 2 °C. Consequently, the different samples were subjected to humidity cycles (HC) between 60% and 100% relative humidity (RH) while imaging. The images recorded at 60% RH after subsequent HC are included in Figure 5. By comparing the fiber mesh evolution over successive HC, it was observed that PVA and PEO already started to lose their fibrous shape after the first cycle, resulting in a completely merged mesh of the liquefied polymer after the second cycle. In contrast, both hydrophilic cross-linked meshes (cPVA and cPEO) and hydrophobic meshes (CA and PCL) showed high stability to HC, with completely unchanged morphology after successive cycles. This was further corroborated by analyzing the fiber diameter change induced by the first HC in all of the samples. As displayed in Figure S5, the fiber diameter distribution remained unaltered after the first HC for all the water-resistant samples (cPVA, cPEO, CA, PCL), while the fiber diameter increased due to a swelling process leading to the dissolution of the water-soluble polymers PVA and PEO.

**Figure 5:**
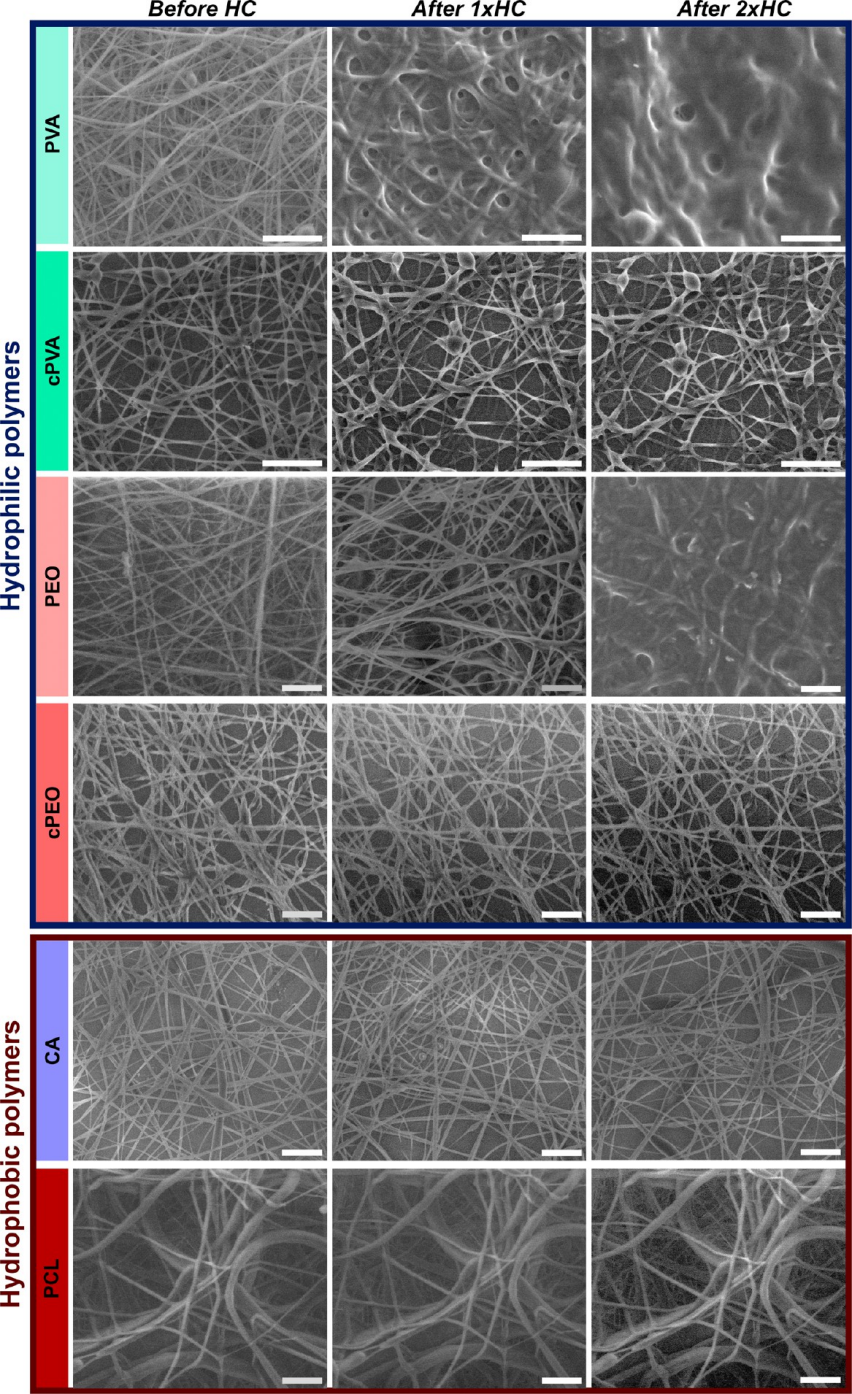
ESEM images of hydrophilic (top) and hydrophobic (bottom) fiber meshes recorded during humidity cycles (HC) between 60% and 100% relative humidity. The fiber meshes were deposited on Si wafers, and the images were recorded at 60% relative humidity after exposure to 100% humidity. The scale bar is 5 μm.

After analyzing the fiber meshes under high-RH conditions, their thermal stability at high temperatures was tested for the final formulations. Thus, the electrospun fibers were exposed to 85 °C for 24 h. Among them, the PCL mesh melted, in accordance with its low melting point (∼60 °C), CA completely retained the mesh structure after heating, while cPVA and cPEO maintained a macroscopic mesh structure but displayed completely merged nanofibers upon closer observation through SEM imaging (Figure 6).

**Figure 6:**
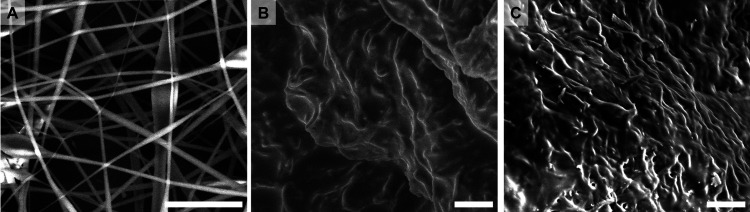
SEM images recorded on CA (a), cPEO (b), and cPVA (c) fiber meshes after heating at 85 °C for 24 h. The scale bar is 5 μm.

To complete the stability assessment of our system, we performed accelerated aging of the samples followed by PCR and sequencing. The accelerated aging was produced by heating at 85 °C for 24 h and subsequently exposing the samples to >97% RH in a sealed chamber for 30 min. The samples were then amplified by PCR and sequenced. The aligned results obtained from Sanger sequencing displayed in Figure 7 showed that successful retrieval of the message after aging was possible for all four samples, even for PCL melted meshes and cPVA and cPEO which lose their nanoscopic fiber structure.

**Figure 7:**
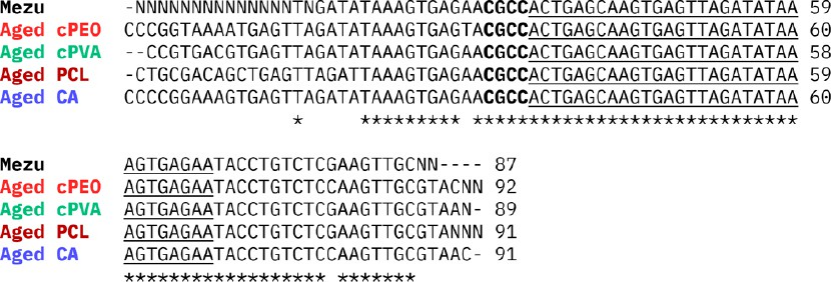
Sanger sequencing results obtained for *Mezu* reference sequence DNA and *Mezu* released from cPVA, cPEO, CA and PCL aged fiber meshes. The sequences obtained were aligned to show the retrieval of the message (underlined) in its second iteration. The 4-bases (CGCC) shown in bold encode a *space* separating the two iterations of the message and are used as a starting point for the decoding process. Overlapping bases among the five sequences are labeled with an asterisk (*).

To evaluate the DNA degradation produced by accelerated aging conditions, qPCR was performed on CA samples, comparing it with the degradation of the standard lyophilized DNA. First, qPCR performed on CA fibers allowed the quantification of the DNA loading for this formulation, which corresponded to 2–5 pg/mg of fibers (Figure S6). Additionally, compared with lyophilized DNA, qPCR quantification shown in Figure S7 displays that the aging conditions caused a 50% degradation for lyophilized DNA and an 80% degradation for CA embedded DNA. This drop in DNA concentration after aging is possibly due to the lower concentration of DNA in the fibers, which could contribute to higher destabilization in accelerated aging conditions.^31^

### Nondestructive Readout (NDR) Memory in CA Fibers

Due to the good performance shown by the CA fiber matrix, we investigated the development of reusable fiber meshes, from which the information could be retrieved multiple times on demand without affecting the fiber structure and stability of the matrix. As previously shown, DNA can be retrieved from CA through water extraction without disrupting the fiber structure due to the hydrophobicity of the polymer. On this basis, we performed a proof-of-concept of an NDR of *Mezu* retrieved from CA fibers multiple times by soaking the sample into water. After the first retrieval, the fiber mesh was dried and stored until the next retrieval cycle, while the water solution used for the extraction was subjected to PCR amplification. Gel electrophoresis (Figure 8A) performed on PCR-amplified samples produced after multiple successive retrieval cycles showed that a constant DNA amount is released from the mesh after each soaking/drying cycle. Additionally, SEM images performed on the fiber mesh after 5 successive soaking/drying cycles demonstrate that the fiber structure remains intact while allowing access to the DNA information in a controlled manner (Figure 8B). Considering the high stability observed for CA fibers, as well as the small amount of DNA that is released in water under the tested conditions (only 7% of the loaded DNA is released after 5 min incubation), we can confidently hypothesize that the matrix can undergo multiple iterative information retrieval cycles.

**Figure 8:**
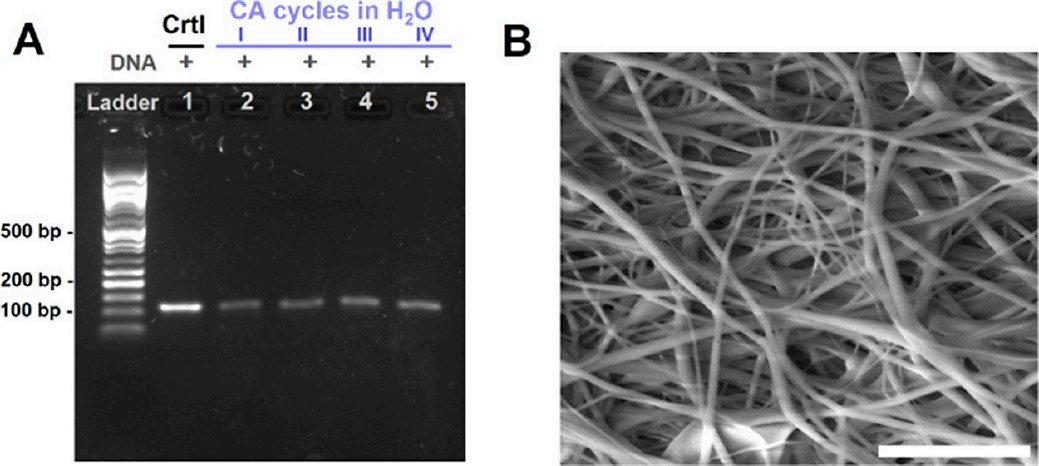
Multiple DNA release cycles from CA fibers. (A) Agarose GE after PCR amplification of original reference DNA sequence (lanes 1) and DNA sequence retrieved from CA after multiple retrieval and soaking/drying cycles in water (lanes 2 to 5). DNA appears as a distinct band in the samples containing DNA (marked as +). (B) SEM image of CA mesh after 5x soaking/drying cycles. The scale bar is 5 μm.

### Storage of Long Messages in a DNA Oligonucleotide Pool (DOP)

Our approach demonstrated the ability to store and retrieve short messages like *Mezu* without degradation in a library of polymeric fibers. To widen the applicability of our system, we extended our encapsulation to the preservation of a longer message encoded in a pool of oligonucleotides instead of a single strand.

In the case of *Mezu*, after the conversion from bits to bases, the message was repeated to protect the information from errors (Figure 9, top). However, for long messages, repeating the entire message within the same strand is not a practical approach due to the current DNA synthesis limitations and information density decrease. In this framework, the Goldman encoding algorithm^32^ is a versatile state-of-the-art method to encode a long message in a pool of several DNA oligonucleotides of controllable length. Here, the input file in bits is converted into a single nucleotide sequence, checking for the presence of homopolymers to avoid long repetitions of the same base, as their presence can hinder PCR amplification and sequencing. The DNA string is then split into fragments of 100 bases, overlapping by 75 bases to achieve high redundancy, and alternate segments are converted into their reverse complements for an additional error correction. Index information is then prepended and appended to each fragment to allow the original data reconstruction during the decoding process. We refer to this set of sequences as DOP (Figure 9, bottom).

**Figure 9:**
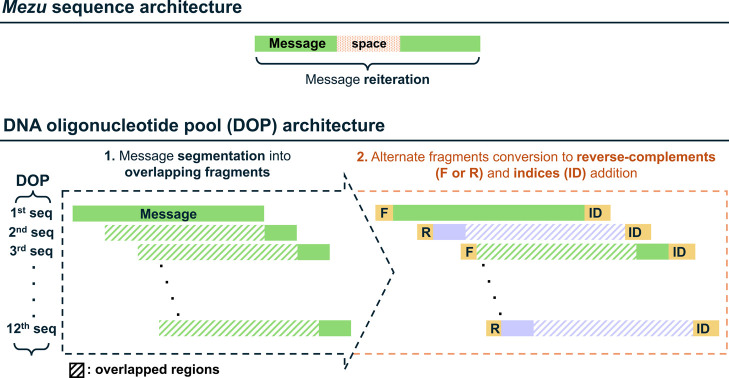
Schematic representation of different sequence architectures for a simple message encoded in *Mezu* (top) and a complex message encoded in DOP (bottom). For *Mezu* redundancy is achieved through message reiteration, separating the two iterations with a *space* (‘**CGCC**’). In the case of DOP, the message is divided into a set of overlapping fragments. Alternate fragments are then converted into their reverse complements, and indices are added to aid in decoding the message upon sequencing.

Using a modified Goldman code (see Materials and Methods), we encoded a 60-byte text and obtained a DOP of 12 sequences, each 92 bases long. The pool was then embedded into various electrospun polymeric fibers, and the DOP retrieval, sequencing, and message decoding were tested to assess the efficiency of the coding scheme in correcting errors introduced during the workflow. In this case, given the complexity of the DOP compared to a single oligonucleotide, the samples were sequenced using Illumina NGS after release and PCR amplification. Following the described workflow, we analyzed the following samples: (i) bare DOP, (ii) PCR amplified DOP, (iii) PCR amplified DOP extracted from PVA, PCL and CA electrospun fibers.

The sequencing results were subjected to clustering and thresholding to select the 12 most represented sequences and then used for decoding. For all analyzed samples, the high throughput of Illumina sequencing enabled the correct selection of the 12 oligonucleotides, which possessed the highest number of reads, with a 10-fold increase in read counts compared to clusters containing erroneous sequences. These results also confirmed that homogeneous PCR amplification of the DOP was possible, not inducing significant amplification bias towards specific oligonucleotides in the pool. Finally, embedding the DOP into different polymeric matrices had no significant effect on sequencing. For all four samples, we successfully retrieved the DNA fragments and decoded them to recover the original message without errors. The complete retrieval of the pool and the decoding of the message showed that the sequencing reads clustering step, and the coding scheme itself could account for errors introduced throughout the workflow. This proof of concept confirmed that electrospun fibers are an effective and promising matrix for safely storing complex, information-bearing DOP.

## Conclusions

In this work, we built a library of electrospinnable water-resistant polymers suitable for midterm DNA digital data storage. The various formulations, including hydrophobic and hydrophilic cross-linked polymers, served as matrices for embedding naked DNA and showed high protection of the oligonucleotide sequence under harsh storage conditions such as high temperature and relative humidity. Aside from an easy production methodology, our electrospun fibers offer a fast workflow for DNA retrieval compared to state-of-the-art methods, with DNA released upon fiber soaking in water or acetone within minutes. Through PCR amplification and sequencing, the message can be recovered and decoded from all the samples, including those subjected to accelerated aging. Additionally, we showed that the outstanding stability of cellulose acetate fibers in aqueous environments allowed the development of a reusable fiber mesh that can undergo several soaking/drying cycles. This mechanism, akin to an NDR memory, allows the repetitive, on-demand retrieval of information without comprosing the fiber structure and integrity. In parallel with developing this polymer library, we took a step further by demonstrating that our storage methodology can be successfully applied to a long message storage encoded in a DOP. Using a modified Goldman code, we successfully retrieved the encoded message from DOP embedded in electrospun fibers employing high-throughput Illumina NGS. The results presented in this work establish an end-to-end workflow as an easy and secure DNA digital storage method, highlighting the versatility of electrospun fibers as a DNA long- and midterm preservation strategy. Further developments of our methodology should address some shared limitations in this research field regarding the message encoding algorithm, including information density and error correction optimization, as well as random-access strategies. Another aspect is completing the characterization of this system and exploring fiber loading limitations in terms of DNA density and sequence length. All these features, combined with our easy, fast, and scalable production and retrieval workflow, pave the way for real-world applications in DNA data storage, such as the inclusion of manufacturing metadata into textiles to improve traceability.

## Materials and Methods

### Materials

Poly­(vinyl alcohol) (PVA, 146–186 kDa, 99+% hydrolyzed), poly­(ethylene oxide) (PEO, average Mv 400 kDa), polycaprolactone (PCL, average Mn 45 kDa), cellulose acetate (average Mn 50 kDa), trimethylolpropane triacrylate (TMPTA, technical grade), benzophenone (99%), and glutaraldehyde (GA, 70% in water) were purchased from Merck. DNA Gel Loading Dye (6x), Analytical-grade Agarose LE and SYBR Safe DNA Gel Stain (10,000x) were purchased from Thermofisher; OneTaq HotStart, HiFidelity polymerase was purchased from New England Biolabs; FastLoad 50 bp DNA Ladder was purchased from SERVA; dNEAT PCR Purification Kit was acquired from Labbox. Oligonucleotides were synthesized and sequenced by Eurofins Genomics (Germany). Sanger sequencing was performed by Stab Vida (Portugal).

### Polymer/DNA Mixture Preparation

#### Poly­(vinyl alcohol)

80 mg of PVA and 10 μL of DNA 100 μM solution were dissolved in water, resulting in final concentrations of 8% (w/w) and 0.0034% (w/w), respectively. Additionally, 0.25% (w/w) of Triton X surfactant was added to the mixture to minimize the surface tension, facilitating the fiber formation process. The solution was stirred overnight to ensure optimal homogenization and was then poured into a 3 mL plastic syringe equipped with a 25-gauge needle and electrospun on aluminum foil (*V* = 10 kV, collector-needle distance = 12 cm, flow rate = 0.45 mL/h). The as-formed fiber mesh was subsequently cross-linked by exposure to glutaraldehyde vapor from a 2.5 M solution for 48 h in a sealed vessel. After the incubation, the excess of GA was removed by filtration with Amicon centrifuge filters (MWCO 10 kDa), and the mesh was dried through lyophilization.^30^

#### Cellulose Acetate

100 mg of CA and 10 μL of DNA 100 μM solution were dissolved in acetone, resulting in final concentrations of 10% (w/w) and 0.0034% (w/w), respectively. The solution was stirred overnight to ensure optimal homogenization and then was poured into a 3 mL plastic syringe equipped with a 25-gauge needle and electrospun on aluminum foil (*V* = 15 kV, collector-needle distance = 12 cm, flow rate = 0.45 mL/h).

#### Poly­(ethylene oxide) and Poly­(ethylene oxide)/TMPTA

80 mg of PEO (8% w/w), 34.3 mg of TMPTA (70:30 = PEO:TMPTA), and 10 μL of DNA 100 μM solution were dissolved in water, resulting in final concentrations of 5% (w/w) for PEO and 0.0034% (w/w) for DNA, respectively. Additionally, 1 mg of benzophenone (1.2 wt % vs PEO) was added as the photoinitiator for the cross-linking reaction. The solution was stirred overnight to ensure optimal homogenization and then was poured into a 3 mL plastic syringe equipped with a 25-gauge needle and electrospun on aluminum foil (*V* = 10 kV, collector needle distance = 12 cm, flow rate = 0.25 mL/h). As a control, PEO/DNA fibers were produced using the same composition without the addition of TMPTA and benzophenone. Photocuring of the TMPTA/PEO–DNA meshes was performed using a Spectroline UV lamp with a 254 nm irradiation wavelength (power: 0.17 A). The meshes were irradiated for 4 h under ambient conditions.

#### PCL

250 mg of PCL and 10 μL of DNA 100 μM solution were dissolved in acetone, resulting in final concentrations of 25% (w/w) and 0.0034% (w/w), respectively. The solution was stirred overnight to ensure optimal homogenization and then was poured into a 3 mL plastic syringe equipped with a 25-gauge needle and electrospun on aluminum foil (*V* = 15 kV, collector-needle distance = 20 cm, flow rate = 0.45 mL/h).

DNA used to produce the fibers was *Mezu* (Ext. Coefficient 1,126,400 L mol^–1^ cm^–1^) or DOP, with sequences included in Table 1. *Mezu* was encoded following the algorithm reported in our previous work.^26^ To encode DOP, we used a modified Goldman algorithm extensively described in the ESI. UV-Vis spectroscopy of DNA solutions was performed with a NanoDrop spectrometer (Thermo Fisher).

**Table 1:**
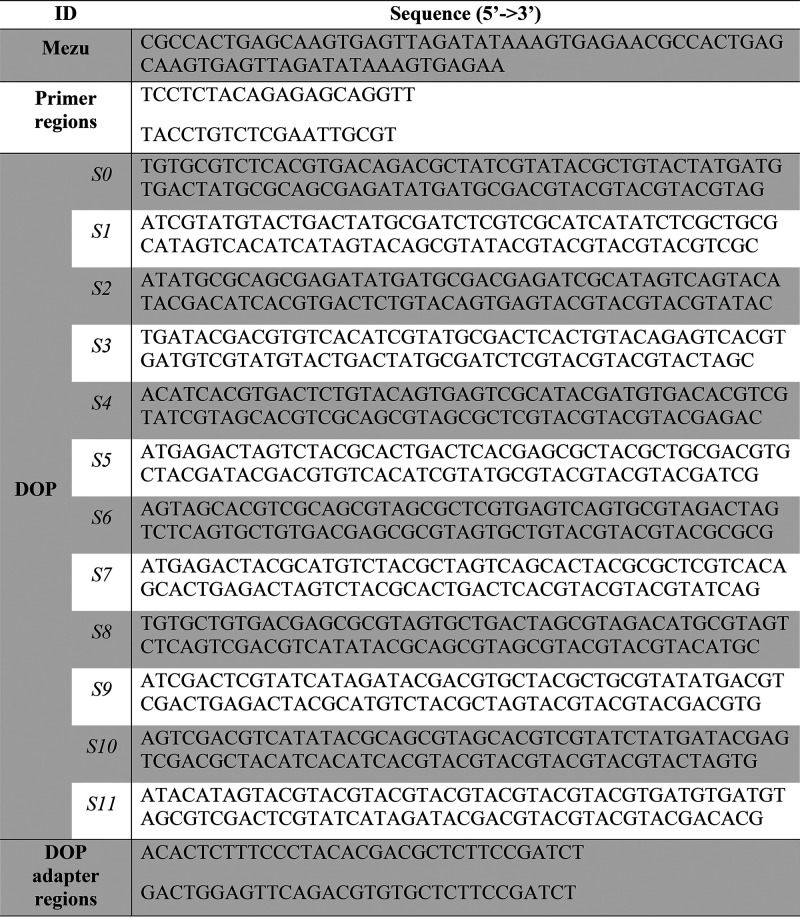
DNA Sequences Encapsulated in Polymeric Fibers

### Electrospinning

Electrospinning was performed by using a E-fiber EF100 electrospinning SKE system. The system was used in a horizontal set up and is composed of a high-voltage power supply, a programmable syringe pump, and a stationary grounded collector. Fiber deposition was performed on aluminum foil or a silicon substrate.

### PCR Amplification

The PCR reactions were carried out in the MiniAmpTM Plus thermal cycler (Applied Biosystems, ThermoFisher). Using the OneTaq HotStart, HiFidelity polymerase, the reactions were performed on 1 ng of DNA template or 5 μL of fibers-DNA mix, with forward and reverse primers at 0.5 μM final concentration in a reaction volume of 50 μL. The PCR reaction cycle started with an initial denaturation at 94 °C for 30 s, followed by 30 cycles of 10 s denaturation at 94 °C, 30 s annealing at 65 °C, 30 s of extension at 68 °C and a final extension at 68 °C for up to 3 min. PCR products were then analyzed by electrophoresis on 2% agarose TBE 0.5X gels, prestained with SYBRSafe according to the manufacturer protocol. For each sample, 5 μL was mixed with 1 μL of Loading Dye (6x) and injected into the gel well for migration at 80 V. A 50 bp molecular weight ladder was used as a reference. Images were recorded with a BioRad UV transilluminator.

### qPCR

qPCR reactions were carried out using a CFX Opus 96 Real-Time PCR System (Bio-Rad). The reactions were performed on 1 μL of DNA samples using 10 μL of iTaq Universal SYBR Green Supermix, with forward and reverse primers at 300 nM final concentration in a reaction volume of 20 μL. A standard thermal protocol was performed according to the manufacturer’s instructions. Relative sample quantification was performed by interpolation from a standard curve generated from standard DNA samples of known concentration. CFX Maestro software version 2.3 (Bio-Rad) was used to perform baseline correction and calculate the threshold cycles (Ct) and relative concentrations.

### DNA Sequencing

Prior to sequencing, PCR amplified samples were purified using a dNEAT PCR Purification Kit (Labbox) according to the manufacturer protocol. Purified DNA samples were sent to Stab Vida (Portugal) for Sanger sequencing and to Eurofins Genomics (Germany) for Illumina sequencing (Novaseq equipment). The resulting fastq files were analyzed by using a Python 3 in-house program.

### SEM and ESEM

Environmental scanning electron microscopy was performed with an FEI Quanta 250. High humidity was obtained by setting the specimen temperature to 2 °C and changing the chamber pressure, producing increasing water condensation.

### Accelerated Aging Tests

Fiber meshes were subjected to high humidity and high temperature to evaluate their stability. Specifically, the fiber meshes were exposed to a >95% water humidity atmosphere for 30 min in a sealed chamber. High-temperature stability was tested by heating the fiber mesh at 85 °C in an oven. Additionally, fiber meshes were exposed to 85 °C and 30 min of high humidity subsequently. All the treated meshed were then analyzed by SEM and PCR amplification/GE.

## Supplementary Material



## Data Availability

The raw sequencing data and generated data sets are available in the ENA (European Nucleotide Archive) repository, Accession Number—ERR15164859.

## Notes

Additional UV–vis spectra, GE and SEM characterization, qPCR results, and sequencing and coding information (PDF)

## References

[1] Reinsel, D. ; Gantz, J. ; Rydning, J. Data Age 2025: The Evolution of Data to Life-Critical Don’t Focus on Big Data; Focus on the Data That’s Big, 2017. www.idc.com.

[2] Qiu T., Chi J., Zhou X., Ning Z., Atiquzzaman M., Wu D. O. (2020). Edge Computing in Industrial Internet of Things: Architecture, Advances
and Challenges. IEEE Communications Surveys
and Tutorials.

[3] Al-Fuqaha A., Guizani M., Mohammadi M., Aledhari M., Ayyash M. (2015). Internet of
Things: A Survey on Enabling Technologies, Protocols, and Applications. IEEE Communications Surveys and Tutorials.

[4] Al-Jaroodi J., Mohamed N. (2019). Blockchain in Industries:
A Survey. IEEE Access.

[5] Andrae A., Edler T. (2015). On Global Electricity
Usage of Communication Technology: Trends to
2030. Challenges.

[6] Monserrate, S. G. The Cloud Is Material: On the Environmental Impacts of Computation and Data Storage; MIT Case Studies in Social and Ethical Responsibilities of Computing, 2022, No. Winter 2022 (January).

[7] Lencer D., Salinga M., Wuttig M. (2011). Design Rules
for Phase-Change Materials
in Data Storage Applications. Adv. Mater..

[8] Rosmeulen, M. ; de la Rosa, C. L. ; Willems, K. ; Fransen, S. ; Shih, B.-Y. ; Verreck, D. ; Kalangi, V. ; Yasin, F. ; Philipsen, H. ; Set, Y. T. ; Ronchi, N. ; Van Roy, W. ; Henry, O. Y. F. ; Arreghini, A. ; Van den bosch, G. ; Furnémont, A. In Liquid Memory and the Future of Data Storage, 2022 IEEE International Memory Workshop (IMW), 2022; pp 1–4.

[9] Zhang, J. ; Čerkauskaitė, A. ; Drevinskas, R. ; Patel, A. ; Beresna, M. ; Kazansky, P. G. In Eternal 5D Data Storage by Ultrafast Laser Writing in Glass, Laser-based Micro-and Nanoprocessing X, 2016; Vol. 9736, p 97360U.

[10] Zhao W., Cai S., Wei X., Zheng T., Xu X., Zafar A., Liu H., Yu T., Lu J., Chen Y., Ni Z. (2021). The Thinnest
Light Disk: Rewritable Data Storage and Encryption on WS2Monolayers. Adv. Funct Mater..

[11] Erlich Y., Zielinski D. (1979). DNA Fountain Enables a Robust and Efficient Storage
Architecture. Science.

[12] Doricchi A., Platnich C. M., Gimpel A., Horn F., Earle M., Lanzavecchia G., Cortajarena A. L., Liz-Marzán L. M., Liu N., Heckel R., Grass R. N., Krahne R., Keyser U. F., Garoli D. (2022). Emerging Approaches
to DNA Data Storage: Challenges
and Prospects. ACS Nano.

[13] Matange K., Tuck J. M., Keung A. J. (2021). DNA Stability:
A Central Design Consideration
for DNA Data Storage Systems. Nat. Commun..

[14] Zhang X., Qi B., Niu Y. (2024). A Dual-Rule
Encoding DNA Storage System Using Chaotic
Mapping to Control GC Content. Bioinformatics.

[15] Ping Z., Chen S., Zhou G., Huang X., Zhu S. J., Zhang H., Lee H. H., Lan Z., Cui J., Chen T., Zhang W., Yang H., Xu X., Church G. M., Shen Y. (2022). Towards Practical and Robust DNA-Based
Data Archiving Using the Yin–Yang Codec System. Nat. Comput. Sci..

[16] Lee H. H., Kalhor R., Goela N., Bolot J., Church G. M. (2019). Terminator-Free
Template-Independent Enzymatic DNA Synthesis for Digital Information
Storage. Nat. Commun..

[17] Slatko B. E., Gardner A. F., Ausubel F. M. (2018). Overview
of Next-Generation Sequencing
Technologies. Curr. Protoc Mol. Biol..

[18] Wang Y., Zhao Y., Bollas A., Wang Y., Au K. F. (2021). Nanopore
Sequencing Technology, Bioinformatics and Applications. Nat. Biotechnol..

[19] Banal J. L., Shepherd T. R., Berleant J., Huang H., Reyes M., Ackerman C. M., Blainey P. C., Bathe M. (2021). Random Access DNA Memory
Using Boolean Search in an Archival File Storage System. Nat. Mater..

[20] Koch J., Gantenbein S., Masania K., Stark W. J., Erlich Y., Grass R. N. (2020). A DNA-of-Things
Storage Architecture to Create Materials
with Embedded Memory. Nat. Biotechnol..

[21] Grass R. N., Heckel R., Puddu M., Paunescu D., Stark W. J. (2015). Robust
Chemical Preservation of Digital Information on DNA in Silica with
Error-Correcting Codes. Angew. Chem., Int. Ed..

[22] Choi Y., Bae H. J., Lee A. C., Choi H., Lee D., Ryu T., Hyun J., Kim S., Kim H., Song S. H., Kim K., Park W., Kwon S. (2020). DNA Micro-Disks for the Management
of DNA-Based Data Storage with Index and Write-Once–Read-Many
(WORM) Memory Features. Adv. Mater..

[23] Prince E., Cheng H. F., Banal J. L., Johnson J. A. (2024). Reversible Nucleic
Acid Storage in Deconstructable Glassy Polymer Networks. J. Am. Chem. Soc..

[24] Bögels B. W. A., Nguyen B. H., Ward D., Gascoigne L., Schrijver D. P., Makri Pistikou A. M., Joesaar A., Yang S., Voets I. K., Mulder W. J. M., Phillips A., Mann S., Seelig G., Strauss K., Chen Y. J., de Greef T. F. A. (2023). DNA
Storage in Thermoresponsive Microcapsules for Repeated Random Multiplexed
Data Access. Nat. Nanotechnol.

[25] Liu Y., Zheng Z., Gong H., Liu M., Guo S., Li G., Wang X., Kaplan D. L. (2017). DNA Preservation
in Silk. Biomater Sci..

[26] Soukarie D., Nocete L., Bittner A. M., Santiago I. (2024). DNA Data Storage in
Electrospun and Melt-Electrowritten Composite Nucleic Acid-Polymer
Fibers. Mater. Today Bio.

[27] Puhl D. L., Mohanraj D., Nelson D. W., Gilbert R. J. (2022). Designing Electrospun
Fiber Platforms for Efficient Delivery of Genetic Material and Genome
Editing Tools. Adv. Drug Deliv Rev..

[28] Legardeur, J. ; Ospital, P. Digital Product Passport in the Textile Sector; Publications Office of the European Union, 2024.

[29] El-Aassar M. R., Elnouby M., Kamal F. H., Badawy N. A., Amer S. I. (2016). Chemical
Crosslinking of Poly (Vinyl Alcohol)/ Polyethylene Glycol with Glutaraldehyde
Nanofibers. Al Azhar Bull. Sci..

[30] Destaye A. G., Lin C. K., Lee C. K. (2013). Glutaraldehyde
Vapor Cross-Linked
Nanofibrous PVA Mat with in Situ Formed Silver Nanoparticles. ACS Appl. Mater. Interfaces.

[31] Baoutina A., Bhat S., Partis L., Emslie K. R. (2019). Storage Stability
of Solutions of DNA Standards. Anal. Chem..

[32] Goldman N., Bertone P., Chen S., Dessimoz C., LeProust E. M., Sipos B., Birney E. (2013). Towards Practical, High-Capacity,
Low-Maintenance Information Storage in Synthesized DNA. Nature.

