# Supplementary material for: Integrating DNA-Based Memory in Water-Resistant Electrospun Polymer Fibers for Nondestructive Data Retrieval
Source: ACS Appl Mater Interfaces. 2025 Jul 30;17(32):46089–98. doi: 10.1021/acsami.5c06554 (PMC12356534; doi:10.1021/acsami.5c06554)
Supplement: Supplementary file 1 [file am5c06554_si_001.pdf]

Supporting information

Integrating DNA-Based Memory in Water-Resistant Electrospun Polymer Fibers for Nondestructive Data Retrieval

Cecilia Wetzl[a], Diana Soukarie[a], Jokin Yeregui Elosua[a,b] and Ibon Santiago[a]*

[a]CIC nanoGUNE BRTA, Donostia-San Sebastián, 20018, Spain

[b]University of the Basque Country, UPV/EHU, Donostia-San Sebastián, 20018, Spain

[*]i.santiago@nanogune.eu

Table of contents

Figure S1. Fiber diameter distribution calculated from SEM images...2

Figure S2. Absorption spectra of polymeric fiber without DNA...2

Figure S3. Additional GE for PCL and CA in water and acetone. 3

Figure S4. Additional Sanger sequencing results 4

Figure S5. Fiber diameter after ESEM experiments..4

Figure S6. qPCR quantification of CA fibers. 5

Figure S7. qPCR quantification on dry DNA and CA fibers before and after aging.....................6

Additional raw data of aligned Sanger sequencing results 7

Additional information on encoding scheme...7

Additional information on sequencing and decoding scheme 9

Figure S1. Fiber diameter distribution calculated from SEM images

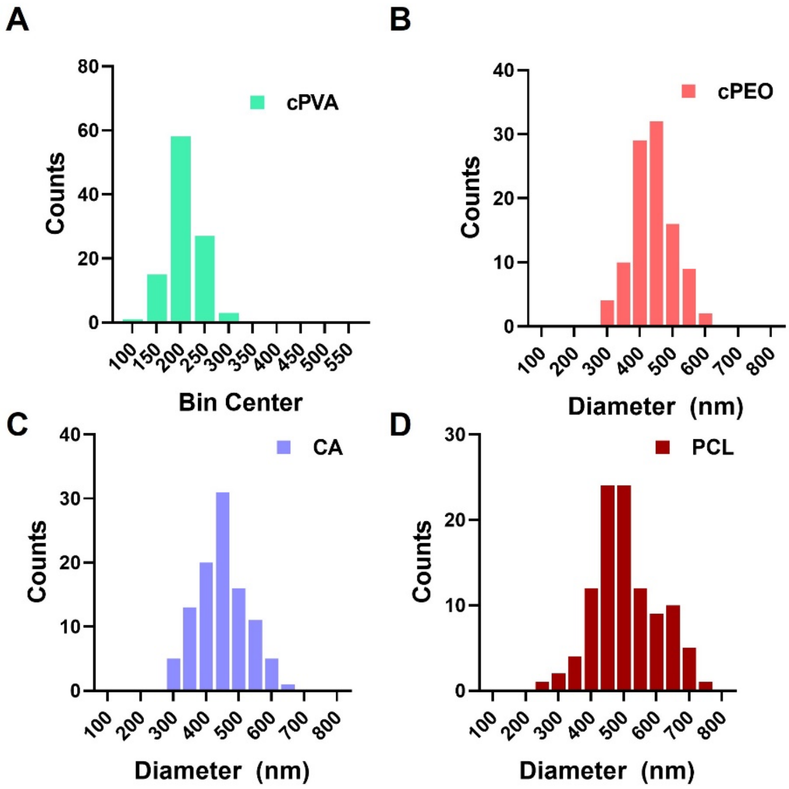

Fiber diameter distributions extracted from SEM images of (a) cPVA, (b) cPEO, (c) CA, and (d) PCL. The histograms represent the frequency distribution of measured fiber diameters for each sample (n=102, bin size= 50 nm).

Figure S2. Absorption spectra of polymeric fiber without DNA

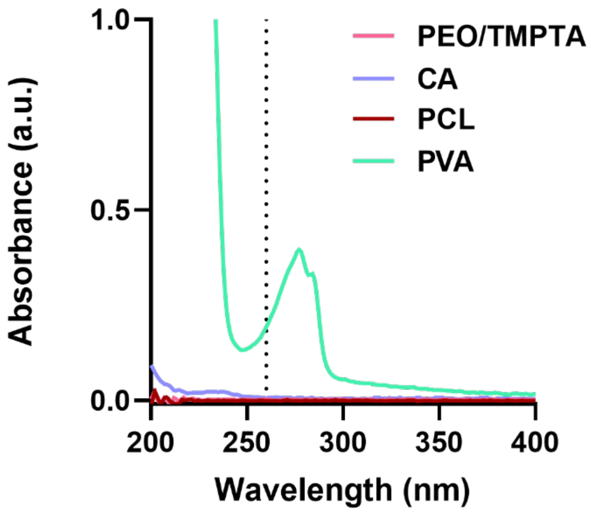

UV-Vis absorption spectra of PEO/TMPTA, CA, PCL, and PVA fibers in water.

Figure S3. Additional GE for PCL and CA in water and acetone

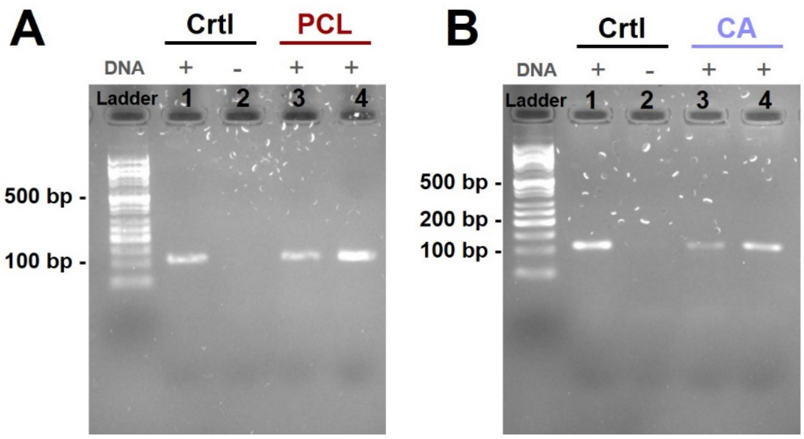

Agarose gel electrophoresis (GE) after PCR amplification of the original reference DNA sequence (lanes 1 and 2) and DNA sequence retrieved from PCL (gel **A**, lanes 3 and 4) or CA (gel **B**, lanes 3 and 4) fibers. Samples in lanes 3 were soaked in water, while those in lane 4 were dissolved in acetone. DNA appears as a distinct band in the samples containing DNA (marked as +), while no nonspecific amplification is detected in samples without reference DNA (marked as -).

Figure S4. Additional Sanger sequencing results

		40	50	60	70	80	
**MEZU**	5′-(…)-GAA**CGCC**ACT	GAGCAAGTGA	GTTAGATATA	AAGTGAGAAT	ACCTGTCTCG-(…)-3′		
**cPVA**	5′-(…)-GAA**CGCC**ACT	GAGCAAGTGA	GTTAGATATA	AAGTGAGAAT	ACCTGTCTCG-(…)-3′		
**cPEO**	5′-(…)-GAA**CGCC**ACT	GAGCAAGTGA	GTTAGATATA	AAGTGAGAAT	ACCTGTCTCG-(…)-3′		
**CA**	5′-(…)-GA-**CGCC**ACT	GAGCAAGTGA	GTTAGATATA	AAGTGAGAAT	ACCTGTCTCG-(…)-3′		
**PCL**	5′-(…)-GAA**CGCC**ACT	GAGCAAGTGA	GTTAGATATA	AAGTGAGAAT	ACCTGTCTCG-(…)-3′		

Sanger sequencing results obtained for the *Mezu* reference DNA and *Mezu* released from cPVA, cPEO, CA, and PCL fiber meshes. The sequences obtained were aligned to show the retrieval of the message (underlined) in its second iteration. The 4-base sequence ‘CGCC’, shown in bold, encodes a bar space separating the two iterations of the message and is used as the starting point for the decoding process.

Figure S5. Fiber diameter after ESEM experiments.

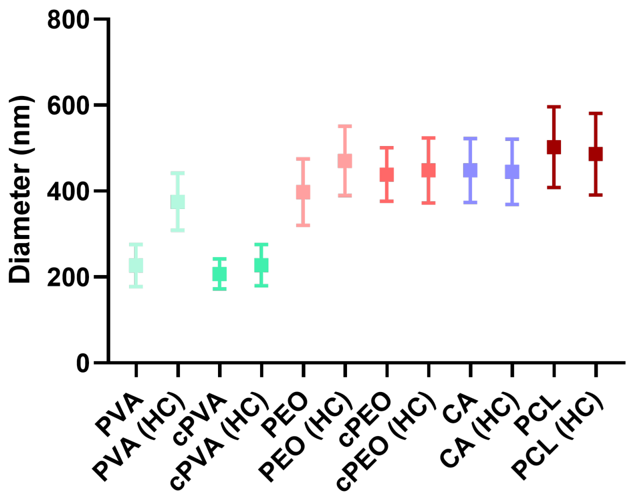

Fiber diameter distribution (n= 102 for all the samples) calculated from SEM for all samples before and after a humidity cycle (HC).

Figure S6. qPCR quantification of CA fibers.

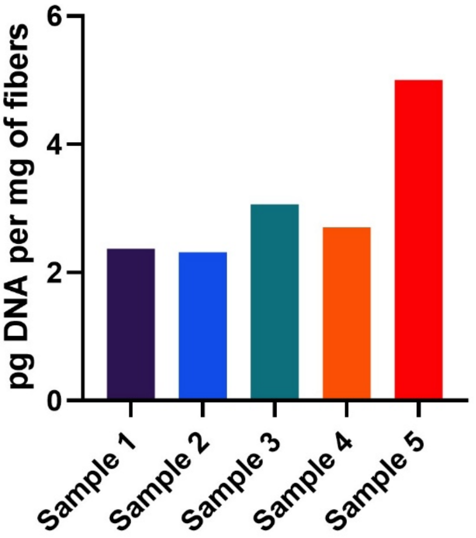

qPCR quantification of DNA loading on different samples of CA fibers. qPCR analysis was performed on 1 μL of fiber solution dissolved in acetone.

Figure S7. qPCR quantification on dry DNA and CA fibers before and after aging.

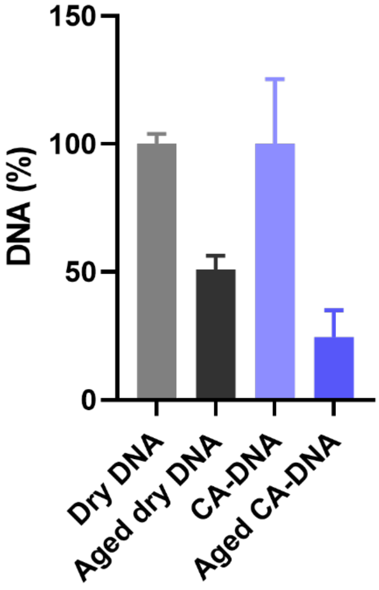

qPCR quantification performed on Dry DNA and CA fibers before and after aging. Accelerated aging was performed exposing the sample to 85 °C for 24h and > 97% RH for 30 minutes. The results are expressed as average ± standard deviation (n=3) and are normalized to the average value obtained for the samples before aging to extrapolate the DNA degradation produced by the aging conditions.

Additional raw data of aligned Sanger sequencing results

```
Mezu -NNNNNNNNNNNNNNTNGATATAAAGTGAGAACGCCACTGAGCAAGTGAGTTAGATATAA 59
CA   ------NNNNNNNNNNNAGATATAAAGTGAGACGCCACTGAGCAAGTGAGTTAGATATAA 54
cPEO ---CCGGTGAGGTGAGTAGATATAAGTGAGAACGCCACTGAGCAAGTGAGTTAGATATAA 57
cPVA CTTGCGACAGGTGAGTTAGATTAAAGTGAGAACGCCACTGAGCAAGTGAGTTAGATATAA 60
PCL  -TTGCGACAGATGAGTTAGAATAAAGTGAGAACGCCACTGAGCAAGTGAGTTAGATATAA 59
                                 ** *****************************

Mezu AGTGAGAATACCTGTCTCGAAGTTGCNN---- 87
CA   AGTGAGAATACCTGTCTCGAAGTTGCGTA--- 83
cPEO AGTGAGAATACCTGTCTCGAAGTTGCGTAANN 89
cPVA AGTGAGAATACCTGTCTCGAAGTTGCGTANN- 91
PCL  AGTGAGAATACCTGTCTCGAAGTTGCGTANN- 90
     **************************
```

```
Mezu      -NNNNNNNNNNNNNNTNGATATAAAGTGAGAACGCCACTGAGCAAGTGAGTTAGATATAA 59
Aged PCL  -CTGCGACAGCTGAGTTAGATTAAAGTGAGAACGCCACTGAGCAAGTGAGTTAGATATAA 59
Aged cPEO CCCGGTAAAATGAGTTAGATATAAAGTGAGTACGCCACTGAGCAAGTGAGTTAGATATAA 60
Aged cPVA --CCGTGACGTGAGTTAGATATAAAGTGAGAACGCCACTGAGCAAGTGAGTTAGATATAA 58
Aged CA   CCCCGGAAAGTGAGTTAGATATAAAGTGAGAACGCCACTGAGCAAGTGAGTTAGATATAA 60
                         *    ********* ******************************

Mezu      AGTGAGAATACCTGTCTCGAAGTTGCNN---- 87
Aged PCL  AGTGAGAATACCTGTCTCGAAGTTGCGTANNN 91
Aged cPEO AGTGAGAATACCTGTCTCCAAGTTGCGTACNN 92
Aged cPVA AGTGAGAATACCTGTCTCGAAGTTGCGTAAN- 89
Aged CA   AGTGAGAATACCTGTCTCCAAGTTGCGTAAC- 91
          ****************** *******
```

Sanger sequencing aligned sequences for all the samples presented in the main text. Overlapping bases shared by all sequences are labelled with an asterisk (*). The alignments were performed using Clustal Omega.

Additional information on encoding scheme

The encoding scheme, adapted from the Goldman algorithm, is available on the repository textaDNA/Jacquard

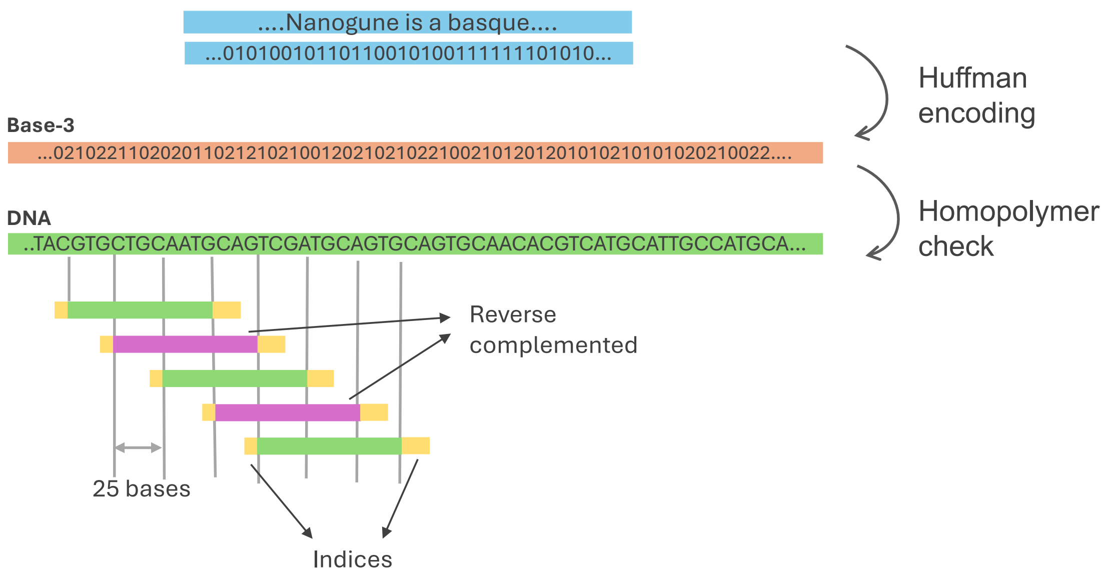

Encoding scheme. A text or binary file (in blue) is converted into base-3 using Huffman encoding (in orange). The second step consists of the actual encoding into nucleotide bases (in green), where the scheme checks for homopolymers. The full strand is then fragmented into 75 base-long oligonucleotides, overlapping by 50 bases and prepended and appended with index bases (in yellow) for a total of 92 bases. Each alternate fragment is reverse-complemented (in purple).

Additional information on sequencing and decoding scheme

A custom-made Python program was used to analyze the FASTQ data obtained from NGS sequencing. A cluster threshold was set in the first preprocessing step to select the clusters with the highest number of reads. The number of selected clusters should correspond to the expected number of fragments to be recovered, as these should be abundantly represented in the sequencing output. Adapters required for Illumina sequencing were then trimmed from the 5′ and 3′ ends of each read. This cleaning step also removed reads with unexpected lengths or those lacking adapters. The decoding process was then run on the selected clusters, with a checksum run in parallel to validate successful decoding and determine whether all fragments can be recovered to reconstruct the original message. The final output is the decoded message.

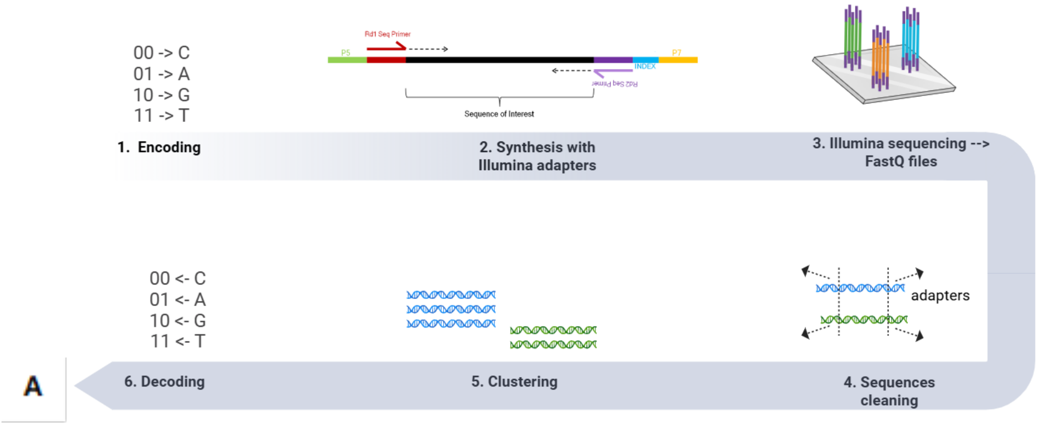

B

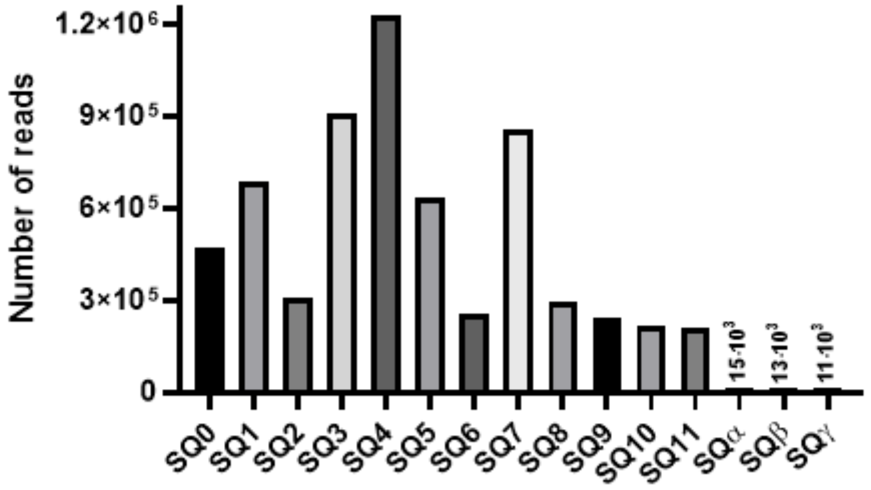

C

ID	Sequence (5'->3')	Reads
**SQ0**	TGTGCGTCTCACGTGACAGACGCTATCGTATACGCTGTACTATGATGTG<br>ACTATG<br>CGCAGCGAGATATGATGCGACGTACGTACGTACGTAG	472425
**SQ1**	ATCGTATGTACTGACTATGCGATCTCGTCGCATCATATCTCGCTGCGCAT<br>AGTCA<br>CATCATAGTACAGCGTATACGTACGTACGTACGTCGC	686548
**SQ2**	ATATGCGCAGCGAGATATGATGCGACGAGATCGCATAGTCAGTACATA<br>CGACA<br>TCACGTGACTCTGTACAGTGAGTACGTACGTACGTATAC	307040
**SQ3**	TGATACGACGTGTCACATCGTATGCGACTCACTGTACAGAGTCACGTGA<br>TGTCG<br>TATGTACTGACTATGCGATCTCGTACGTACGTACTAGC	906838
**SQ4**	ACATCACGTGACTCTGTACAGTGAGTCGCATACGATGTGACACGTCGTA<br>TCGTA<br>GCACGTCGCAGCGTAGCGCTCGTACGTACGTACGAGAC	1226487
**SQ5**	ATGAGACTAGTCTACGCACTGACTCACGAGCGCTACGCTGCGACGTGCT<br>ACG	633302

	ATACGACGTGTCACATCGTATGCGTACGTACGTACGATCG	
**SQ6**	AGTAGCACGTCGCAGCGTAGCGCTCGTGAGTCAGTGCGTAGACTAGTCT<br>CAG<br>TGCTGTGACGAGCGCGTAGTGCTGTACGTACGTACGCGCG	257377
**SQ7**	ATGAGACTACGCATGTCTACGCTAGTCAGCACTACGCGCTCGTCACAGC<br>ACTG<br>AGACTAGTCTACGCACTGACTCACGTACGTACGTATCAG	858057
**SQ8**	TGTGCTGTGACGAGCGCGTAGTGCTGACTAGCGTAGACATGCGTAGTCT<br>CAGT<br>CGACGTCATATACGCAGCGTAGCGTACGTACGTACATGC	297856
**SQ9**	ATCGACTCGTATCATAGATACGACGTGCTACGCTGCGTATATGACGTCG<br>ACTGA<br>GACTACGCATGTCTACGCTAGTACGTACGTACGACGTG	242328
**SQ10**	AGTCGACGTCATATACGCAGCGTAGCACGTCGTATCTATGATACGAGTC<br>GACG<br>CTACATCACATCACGTACGTACGTACGTACGTACTAGTG	214545
**SQ11**	ATACATAGTACGTACGTACGTACGTACGTACGTACGTGATGTGATGTAG<br>CGTCG<br>ACTCGTATCATAGATACGACGTACGTACGTACGACACG	209161
**SQα**	ACATCACGTGACTCTGTACAGTGAGTCGCATACGATGTGACACGTCGTA<br>TCGTA<br>GCACGTCGCAGCGTAGCGCTCGTACGTACGTACGTCGC	15179
**SQβ**	ACATCACGTGACTCTGTACAGTGAGTCGCATACGATGTGACACGTCGTA<br>TCGTA<br>GCACGTCGCAGCGTAGCGCTCGTACGTACGTACGATCG	13159
**SQγ**	ATCGTATGTACTGACTATGCGATCTCGTCGCATCATATCTCGCTGCGCAT<br>AGTCA<br>CATCATAGTACAGCGTATACGTACGTACGTACGAGAC	11620

A. Sequencing and decoding pipeline. 1) Encoding using an adapted Goldman scheme 2) Synthesis of the oligonucleotide pool with additional Illumina adapters. 3) The samples are sequenced using Illumina NovaSeq, producing FastQ files ready for in silico processing. 4) The sequences are trimmed to remove adapters and discard oligos with unexpected lengths. 5) The fragments are clustered. 6) The decoding step of the modified Goldman scheme is run to retrieve the original message. **B.** Example of the DNA Oligo pool (DOP) after PCR sequencing. Number of reads obtained for different clusters after the cleaning step. The most represented clusters correspond to the twelve sequences of the DOP (SQ0 to SQ11), while the first sequences with errors (SQα, SQβ, SQγ) present 100-fold decrease in number of reads, facilitating the clustering step and sequence selection. **C.** Representative clusters after the cleaning step, corresponding to the twelve sequences of the pool. The first errors (highlighted in red) appear in clusters with fewer than 16,000 reads.
